# Study of the Sedimentation Characteristics of Solids in Carwash Wastewater

**DOI:** 10.1002/wer.70281

**Published:** 2026-01-19

**Authors:** João Paulo Cruvinel Miranda, Antônio Alves Martins, Andriane de Melo Rodrigues, Celsio Assane, Édio Damásio da Silva Júnior

**Affiliations:** ^1^ Goiano Federal Institute for Education, Science and Technology Rio Verde Brazil

**Keywords:** granulometry, sand, settleable solids, settling test

## Abstract

Studies evaluating the sedimentation of solid particles in carwash wastewater (CWW) are scarce. This research is innovative because it is the first to study solid sedimentation specifically in CWW. The motivation lies in the fact that existing parameters (for sanitary sewage) are inadequate due to the peculiar physicochemical characteristics of CWW. This study evaluated the settleability of solids present in CWW, aiming to generate empirically validated parameters to support the optimized design of sedimentation units. Granulometric characterization of the settleable material and column settling tests for total suspended solids (TSS) were performed. The granulometric analysis of the settleable solids revealed a predominance of the sandy fraction (D_90%_ = 1.1 mm), with an average of 87.44%. This characteristic confirms the coarse texture of the retained material and its high sedimentation velocity during the first hour. The column settling tests for TSS demonstrated highly variable removal efficiency, which did not directly correlate with the initial concentration of solids or with rainfall conditions. Results indicated the need for hybrid sedimentation models to adequately represent TSS sedimentation. A surface application rate of 1.5 m·h^−1^ is suggested, which corresponds to an average TSS removal efficiency of approximately 80%. The adoption of specific design parameters for CWW provides greater reliability in the sizing of treatment units, supporting both operational efficiency and the economic viability of the system.

## Summary

- Carwash wastewater solids are predominantly sandy (87.44%), indicating coarse texture and rapid sedimentation potential.
- Sedimentation unit designs should not rely solely on sanitary sewage parameters for CWW treatment.
- Column settling tests showed variable TSS removal, independent of initial concentration or rainfall conditions.
- A surface application rate of 1.5 m·h^−1^ achieves ~75% average TSS removal efficiency.
- Empirical data support optimized sedimentation design, improving the reliability of CWW treatment systems.

## Graphical abstract

This study evaluated solids sedimentation in carwash wastewater, highlighting the predominance of sand. A surface application rate of 1.5 m·h^−1^ was proposed to optimize settler design, achieving ~75% solids removal.

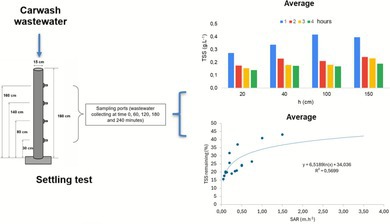

## 1 Introduction

The expansion of the vehicle fleet in urban centers has driven an increase in carwash stations, which are significant sources of water pollution. These establishments generate expressive volumes of carwash wastewater (CWW), a complex matrix effluent that typically features high concentrations of pollutants such as heavy metals, oils and greases, anionic surfactants, and total suspended solids (TSS) (Espinoza‐Montero et al. 2023; Talebzadeh et al. 2021; Cuput et al. 2024). When discharged without proper treatment into the soil, water bodies, or the sanitary sewer system, these effluents can cause significant environmental, sanitary, and economic impacts, compromising public health and the balance of aquatic ecosystems.

Considering the high levels of pollutants present in CWW and its potential impacts, it is imperative that the treatment process is effective, ensuring the removal of key contaminants and meeting the quality standards required by current environmental legislation.

Processes and technologies reported in the literature for this purpose generally include coagulation‐flocculation, electrocoagulation, flotation, membrane separation, and advanced oxidation, each with varying degrees of efficiency in removing specific pollutants and associated operational costs (Moazzem et al. 2020; Veit et al. 2020; Kuan et al. 2022). However, the technical and economic viability of such processes fundamentally depends on the effectiveness of a preliminary stage for removing larger solids in sedimentation units, such as a grit chamber. The prior removal of settleable solids is essential to reduce operational costs and improve the quality of the wastewater to be purified in subsequent stages, in addition to preventing clogging and premature wear of the equipment used in sequential treatment processes (Plana et al. 2018).

Dissolved solids and those with low settling capacity can be removed through filtration, coagulation‐flocculation, or membrane separation processes, whereas solids with high settleability require units like grit chamber and settling tanks (clarifiers), which can promote efficient gravitational separation of these materials (Veit et al. 2020; Alazaiza et al. 2024; Silva Júnior et al. 2025). Depending on the particle size distribution (clay, silt, sand, or gravel), CWW will present distinct treatment demands, requiring specific approaches to remove particles according to their density, shape, and hydrodynamic behavior.

The complexity of treating CWW is accentuated by the variability of its characteristics. The content and nature of the solids can vary drastically depending on factors like the predominant soil type in the region. Additionally, the concentration of settleable solids in CWW can be strongly influenced by rainfall rates. During intense rain events, mud forms on public roads due to the surface runoff of soil particles, urban waste, and accumulated particulate matter (Pinto et al. 2017; Monney et al. 2020). Consequently, vehicles travel in dirtier environments, resulting in the generation of liquid effluents with a higher load of settleable solids during the washing process. This significantly increases the demand for physical separation processes and raises the risk of overloading the treatment units (Tchobanoglous et al. 2014).

During dry periods, CWW tends to have a lower concentration of settleable solids compared to the rainy season. Given this variability, treatment units must be designed to operate efficiently under both low and high solid concentration conditions, which requires in‐depth knowledge of the settling behavior of particles in different operational scenarios.

Solid particle sedimentation is assumed to be a complex procedure in water and wastewater treatment plants. There is great interest in applying and developing different simulation and optimization methods to design primary sedimentation tanks (Zamanikherad et al. 2023). The design of sedimentation units is traditionally based on established physical principles, governed by the settling velocity of particles, a parameter that defines the surface application rate (SAR) and the hydraulic retention time (HRT) (Von Sperling 2014). Classical models assume that suspended particles exhibit discrete settling (type 1) or, more commonly, flocculant settling (type 2), where the aggregation of smaller particles favors their removal by gravitational action (Tchobanoglous et al. 2014).

However, the direct application of this paradigm to CWW constitutes a technically limited extrapolation. The heterogeneous and highly variable nature of CWW (influenced by factors such as soil granulometry, rainfall intensity, and the presence of organic and inorganic contaminants) demands specific approaches that consider the physicochemical complexity of these effluents.

The physicochemical composition of CWW can influence the fundamental premises of classical sedimentation models. The presence of high concentrations of anionic surfactants, for example, due to their amphiphilic nature, can actively inhibit the aggregation (flocculation) process. These compounds stabilize suspended oil droplets and adsorb onto the surface of solid particles, imparting a surface charge and steric hindrance (Yang et al. 2021; Zhang et al. 2025). As a result, the system ceases to behave as a flocculant suspension and begins to resemble a stabilized colloidal suspension condition for which conventionally designed models, based exclusively on hydraulic parameters, become intrinsically invalid.

This research lies in the pioneering and essentially unprecedented approach to studying the sedimentation and behavior of solid particles in CWW. Although the scientific literature contains robust studies on solid sedimentation in sanitary sewage or other industrial effluents, such models and parameters are inadequate for CWW. This is due to the peculiar physicochemical characteristics of this effluent, which contains a specific mixture of oils, greases, detergents, and abrasive particles derived from vehicle cleaning, in contrast to the predominantly organic composition of sanitary sewage. Thus, by generating empirically validated and specific parameters for the settleability of solids in CWW, this research not only fills a critical gap in technical‐scientific knowledge but also provides the necessary foundation for the optimized and more efficient design of treatment units, maximizing pollutant removal and the environmental sustainability of the activity.

Given this scenario, the present study aims to evaluate the settleability of solids in CWW to generate empirically validated criteria that can support the optimized engineering of these treatment units.

## 2 Materials and Methods

### 2.1 Study Area

This study was conducted at a commercial vehicle wash facility located in the municipality of Rio Verde, in the central region of Brazil (latitude: 17°47′45.5″S; longitude: 50°54′59.9″W). The region is characterized by a humid tropical climate with well‐defined seasonality. The summer period, from October to March, is marked by frequent rains and average temperatures of 25°C, while the winter, from May to August, is predominantly dry, with average temperatures of 21°C. The average annual rainfall is 1493 mm.

This facility employs a mixed washing system, combining automatic roller (rollover) technology and manual high‐pressure water jets, in addition to offering complementary services such as wax applications. In the cleaning process, alkaline detergent solutions and chemical products are used, such as degreasers, multipurpose cleaners, and synthetic waxes.

The establishment services approximately 400 small and medium‐sized vehicles per week, including cars, motorcycles, and pick‐ups. The water used in the vehicle‐washing processes is groundwater, with an estimated average daily consumption of 30.6 ± 5.56 m^3^. After washing, the generated effluent is directed through channels to an existing preliminary treatment system, composed of a grit chamber and subsequently, an inspection chamber, before being discharged into the public sanitary sewer system.

### 2.2 Particle Size Analysis of Settleable Solids

**FIGURE 1:**
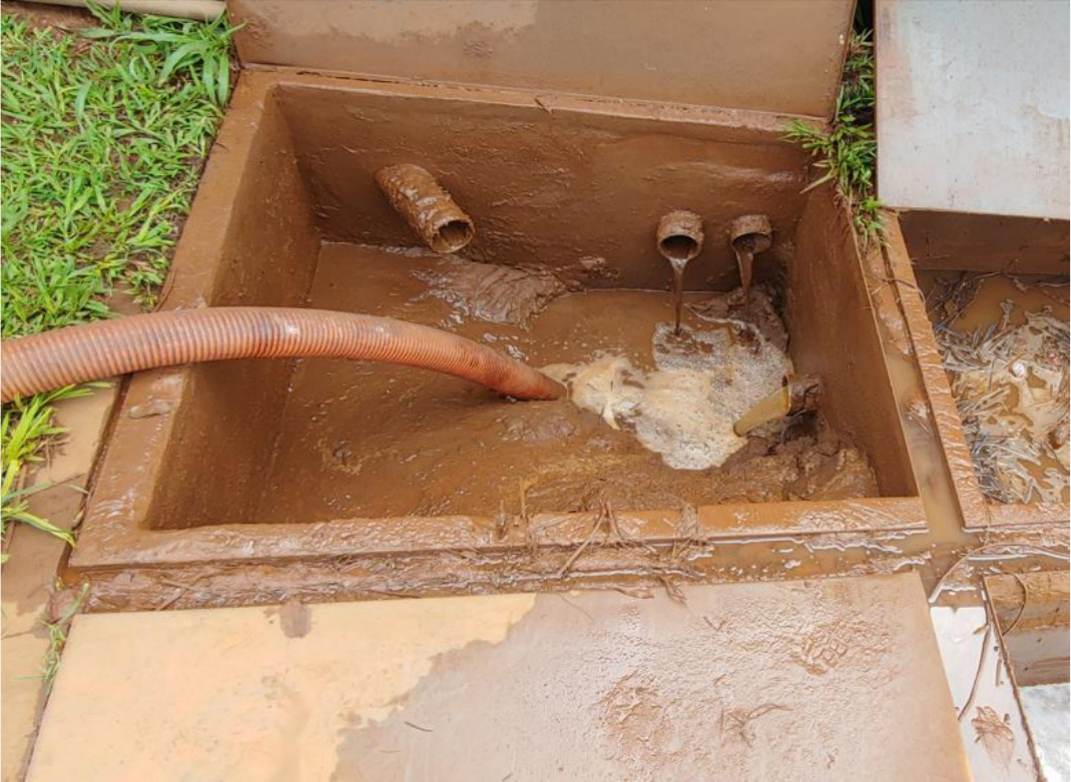
Cleaning of the grit chamber at the vehicle wash facility. The accumulated solid material was evaluated for its particle size distribution.

The granulometric characterization of the settleable solids was conducted to determine the distribution of the constituent fractions of the material accumulated in the grit chamber. For this purpose, real samples (*in natura*) of the material retained in the facility's grit chamber were collected in February 2025, a period corresponding to the region's rainy season. The collection was carried out during a routine cleaning operation of the unit, as illustrated in Figure 1.

The analyses followed the combined sieving and sedimentation method, in accordance with the procedures established by NBR 7181 standard (ABNT 2016), allowing for the determination of particle size distribution and the classification of particles according to their settling behavior. The Brazilian standard NBR 7181 is equivalent to ISO 11277. This is the standard procedure applied for particle size determination, being employed in the analysis of sand particles and solid materials in wastewater.

Initially, the collected samples were subjected to oven‐drying at 105°C–110°C (model TH 510‐480) until it reached a constant mass, with the objective of removing moisture. Subsequently, the dry material was carefully disaggregated to break up clumps while preserving the integrity of the individual particles. From this material, a representative sample was obtained using the quartering method.

**FIGURE 2:**
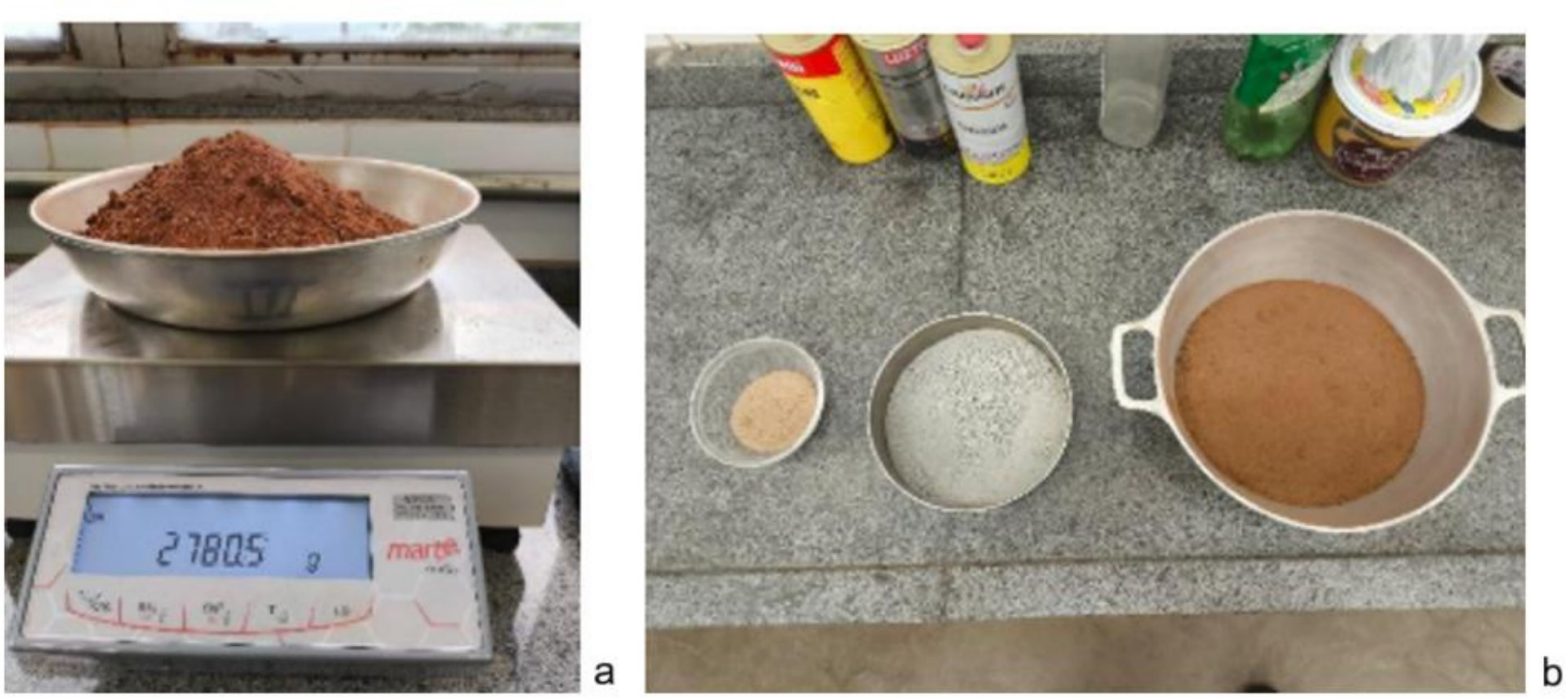
Granulometric analysis of the settleable solids collected in the grit chamber. After oven‐drying (a) and after deflocculation, drying, and sieving (b).

For coarser fractions, such as sand (particles larger than 0.075 mm), the sieving method was used, which required a shaker set (model MBL AGMAGB) with standardized mesh sieves of 0.25, 0.42, and 0.075 mm, a precision balance (model AD10K) to weigh the material retained in each sieve, and an oven for sample drying. The retained fraction in sieves 0.075 mm, corresponding to the sandy portion, was again oven‐dried and subjected to sieving in a series of standard mesh sieves (50–2.0 mm). The mass of the material retained on each sieve was recorded to determine the particle size distribution of the coarse fraction. Figure 2 shows the raw material (after oven‐drying) and the separate fractions obtained at the end of the analytical procedures.

The fraction of material passing through the 0.075‐mm sieve, predominantly composed of silt and clay, was collected in its entirety to perform the sedimentation analysis. This test, based on Stokes' Law, was conducted by dispersing a known mass of fines in a 1000‐mL graduated cylinder containing distilled water and sodium hexametaphosphate as a deflocculating agent, to ensure the individual suspension of particles (ABNT 2016). After complete homogenization, the suspension was left undisturbed, and density readings were taken with a soil densimeter (model Incoterm 5846‐4) at predetermined time intervals.

The integration of the results obtained from the sieving and sedimentation steps allowed for the construction of the complete particle size distribution curve of the material, enabling the quantification of the percentages of sand, silt, and clay, according to the size limits established by the DNIT 198/2021 standard (DNIT 2021).

### 2.3 Column Settling Tests

**FIGURE 3:**
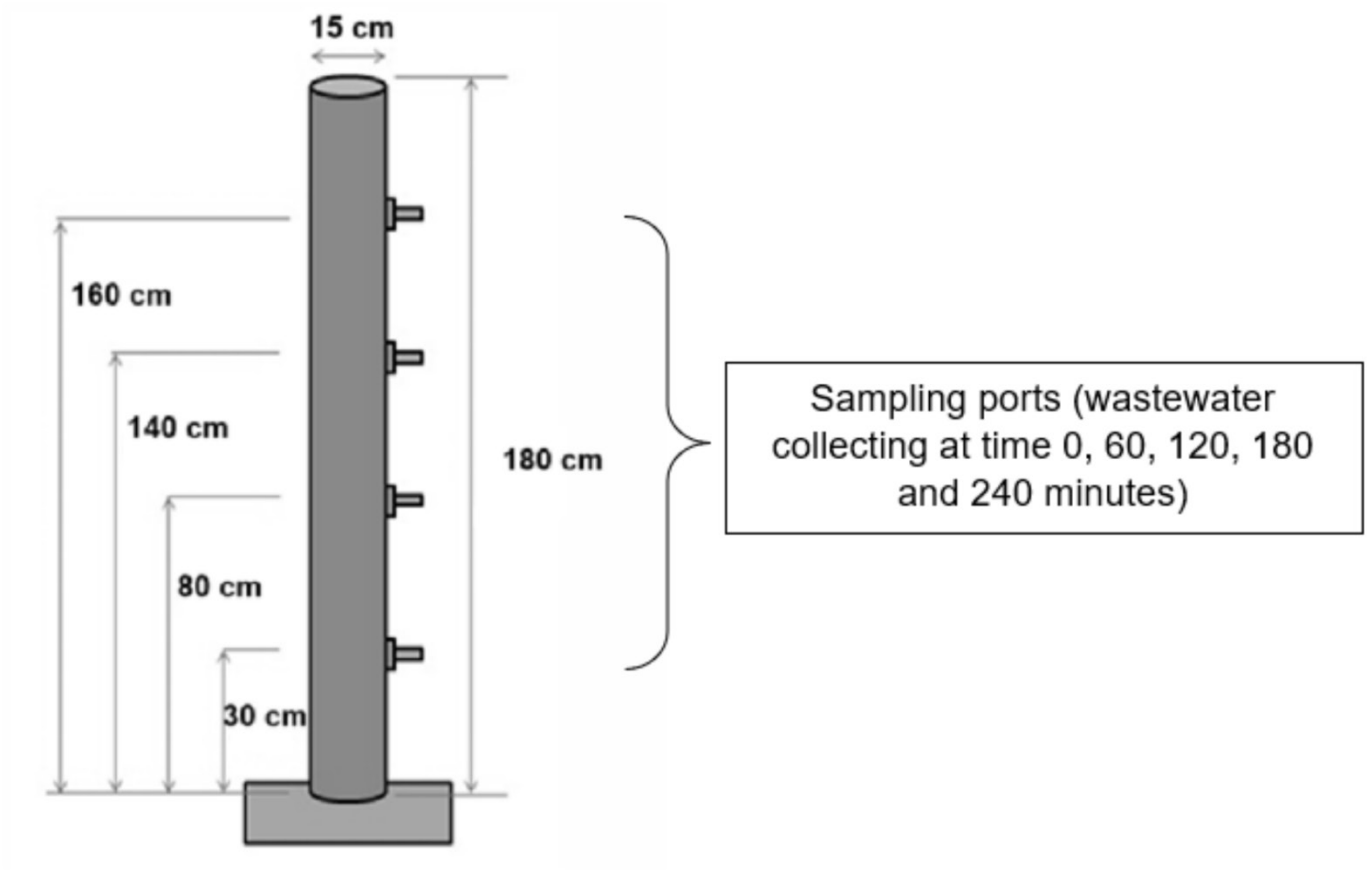
Schematic of the apparatus used for the solids settling test and the experimental stages.

The sedimentation kinetics were evaluated based on the premise of discrete particle settling (type 1), which is characterized by the settling of particles that do not interact or aggregate during the process, thus maintaining constant size, shape, and mass (Tchobanoglous et al. 2014). The tests were conducted in a vertical settling column (Figure 3), made of polyvinyl chloride (PVC), with an effective height of 180 cm and an internal diameter of 15 cm, equipped with four sampling points (taps) positioned at 20, 40, 100, and 150 cm from the top.

There is no established standard in the technical literature for the height of the sedimentation column in bench‐scale tests. Tchobanoglous et al. (2014) indicate that a column can be of any diameter but should be equal in height to the depth of the proposed sedimentation tank. For this study, a value of 180 cm was adopted as a practical approach. This depth is commonly found in grit chamber units and sedimentation tanks in CWW treatment systems. The choice of this value simulates real operating conditions while avoiding the implementation and operational difficulties associated with deeper units, such as the risk of hitting the water table during larger excavations and the increased complexity in the cleaning and accumulated solids removal stages.

For the CWW settling tests, the initial step consisted of obtaining representative samples of the real effluent generated by the facility during full operation. A composite sampling procedure was adopted to minimize the temporal variability of the solids concentration and to obtain a sample that reflected the average conditions of a typical carwash cycle. The procedure involved collecting 500 mL of CWW every 5 min directly from the inlet pipe of the grit chamber. Eight sequential collections were carried out over 35 min, totaling a final volume of 40 L, which was stored in a polyethylene container. Immediately after collection, a fraction of this volume was reserved, in duplicate, for the physicochemical characterization of the raw effluent (time *t* = 0), serving as a reference for the initial concentration of TSS.

For each test, the 40‐L composite sample was vigorously homogenized to ensure the uniform distribution of TSS. Subsequently, the column was filled quickly and continuously to minimize the premature settling of particles. The initial time (*t* = 0) was defined when the column reached its full capacity.

Samples of 100 mL were collected in duplicate from each of the four sampling points at intervals of 1, 2, 3, and 4 h.

To evaluate the influence of rainfall seasonality on the effluent's characteristics, the set of settling tests was replicated five times over 2 months (February and March 2025).

To correlate the results with climatic conditions, an automatic rain gauge station (Ciclus PRF‐1S), installed at the geographic coordinates (latitude: 17°48′38.25″S; longitude: 50°54′52.22″W), was used to record the accumulated precipitation in the 7 days preceding each round of tests.

### 2.4 Analysis of TSS

TSS concentration in all collected samples was performed in duplicate, following gravimetric method 2540 D, as described in the *Standard Methods for the Examination of Water and Wastewater* (APHA 2022). The procedure consisted of vacuum filtering a known volume of the sample (100 mL) through a glass fiber filter with a nominal pore size of 2.0 μm. Before filtration, each filter was previously washed, dried in an oven at 103°C–105°C (model TH 510‐480), cooled in a desiccator, and weighed on a high‐precision analytical balance (model Shimadzu ATY224R) to determine the initial mass. After the sample was passed through, the filter containing the retained material was dried again under the same conditions until it reached a constant mass. The TSS concentration, expressed in milligrams per liter (mg L^−1^), was calculated by the difference between the final and the initial masses of the filter, divided by the volume of the filtered sample.

### 2.5 Data Evaluation

The processing of the experimental data began with the conversion of TSS concentrations into percentage removal values to normalize the results relative to the initial condition. For each sampling point, defined by a depth (*h*) and a time (*t*), the removal efficiency *R* (%) was calculated according to Equation (1), where *C*_0_ represents the initial TSS concentration in the raw effluent and *C*_*h,t*_ corresponds to the concentration measured in the respective sample.

(1) $$R\;\left(\%\right)=\frac{C_{0}-C_{h,t}}{C_{0}}\times 100$$

Isolines of TSS removal efficiency were estimated as a function of sedimentation time and column depth, employing the linear interpolation technique. This approach enabled the determination of the exact coordinates (time and depth) at which a constant and predefined removal efficiency occurred between discrete measurement points, providing a clear and continuous visual representation of sedimentation performance across the time–depth domain.

## 3 Results and Discussion

### 3.1 Particle Size Characterization

The importance of the preliminary sedimentation unit for CWW in removing larger‐sized solids has been emphasized by Torkashvand et al. (2022). Without the preliminary sedimentation stage (e.g., grit chambers), the high concentration of larger‐sized solids may cause damage or compromise the operation of subsequent treatment processes: coagulation‐flocculation, conventional filtration, membrane separation, and advanced oxidation (Plana et al. 2018; Veit et al. 2020; Kuan et al. 2022; Torkashvand et al. 2022).

The results of the particle size analysis of the solids retained in the grit chamber of the studied CWW treatment system are presented in Table 1. It should be noted that the obtained results do not represent the particles in their natural state, but rather after undergoing the granulometric characterization procedures described in the methodology, including the deflocculation step.

**TABLE 1:** Particle size distribution of settleable solids from the grit removal unit.

Portion	Sample 1 (%)	Sample 2 (%)	Sample 3 (%)	Mean (%)	SD
Sand (0.060–2.0 mm)	87.82	85.60	88.91	87.44	1.70
Silt (0.002–0.060 mm)	3.00	1.08	0.72	1.60	1.22
Clay (< 0.002 mm)	4.81	3.85	4.44	4.37	0.49
Silt + clay (< 0.060 mm)	7.81	4.93	5.16	5.97	1.57
D_10%_ (mm)	0.15	0.13	0.17	0.15	0.01
D_50%_ (mm)	0.30	0.34	0.32	0.32	0.02
D_90%_ (mm)	1.1	1.1	1.0	1.1	0.01

A significant predominance of the sand fraction was observed (average 87.44%), followed by silt + clay (average 5.97%), with dimensions smaller than 2.0 mm. The particle size analysis of the solids retained in the grit chamber revealed a median diameter (D_50%_) of 0.32 mm, with D_10%_ equal to 0.15 mm and D_90%_ equal to 1.1 mm. These values indicate a relatively wide particle size distribution, encompassing both fine and coarse fractions. In terms of sedimentation properties, finer particles (around D_10%_) exhibit lower settling velocities and a greater tendency to remain in suspension, whereas coarser particles (around D_90%_) settle rapidly, contributing to the initial removal of solids. The intermediate fraction represented by D_50%_ suggests predominant particle dimensions associated with moderate settling rates, reinforcing the need for hybrid sedimentation models to adequately represent system behavior and ensure treatment efficiency.

Studies on particle size distribution in grit chambers of municipal sewage have also indicated a predominance of particles smaller than 2.0 mm (Borges et al. 2015; Czop et al. 2025). Based on laboratory tests carried out on washed mineral waste recovered from grit chambers in sewage treatment plant, Kostrzewa et al. (2024) concluded that the values of the determined parameters coincide with the values of the geotechnical parameters for sands. The retained material has a coarse texture, characteristic of sediments with a high settling velocity, which is compatible with the operating principle of pretreatment units designed for the removal of settleable solids.

Sand particles are, by nature, granular, noncohesive and possess high density, which predisposes them to settle as individual entities. This process is defined as discrete or type 1 settling, in which the settling velocity of each particle is constant and governed primarily by its intrinsic properties (size, shape, and density) and by the properties of the fluid, in accordance with the principles of classical fluid dynamics (Tchobanoglous et al. 2014).

The high proportion of sand suggests that the main contribution of solids stems from the entrainment of mineral particles of geogenic origin, such as dust and soil fragments adhered to vehicles, as well as abrasive residues from mechanical cleaning processes.

The complexity of CWW treatment is accentuated by the variability of its characteristics. The nature and concentration of solids can vary drastically, being influenced by factors such as the predominant soil type in the region and precipitation rates. During heavy rainfall events, the formation of mud on public roads is intensified due to surface runoff carrying soil particles, urban residues, and accumulated particulate matter (Pinto et al. 2017; Monney et al. 2020). This extreme variability imposes significant challenges to the stability and efficiency of both pretreatment and subsequent treatment processes.

The low fraction of silt and clay indicates that fine particles tend to remain in suspension and are not efficiently removed at this stage, migrating to subsequent treatment units. This behavior is consistent with Stokes' settling theory, according to which particles with a diameter of less than 0.060 mm have significantly lower settling velocities, requiring longer HRTs or complementary processes, such as flotation or filtration, for their removal (Von Sperling 2014).

The degree of resource utilization of solid waste generated by sewerage systems in developing countries is very limited. Based on the idea of sustainable development, it is necessary to expand the methods of resource recovery (Sun et al. 2023).

From an environmental perspective, the particle size characterization informs the proper management of the waste. The sand fraction, when free of hazardous contaminants, may have the potential for reuse in applications such as pavement base or sub‐base, thus reducing its disposal in landfills (Borges et al. 2015; Kostrzewa et al. 2024; Czop et al. 2025; Łaźniewska‐Piekarczyk and Czop 2025). However, the presence of fine particles, even in small amounts, reinforces the need for additional contaminant analyses to assess the risks and feasibility of reuse.

### 3.2 Settling of TSS

**FIGURE 4:**
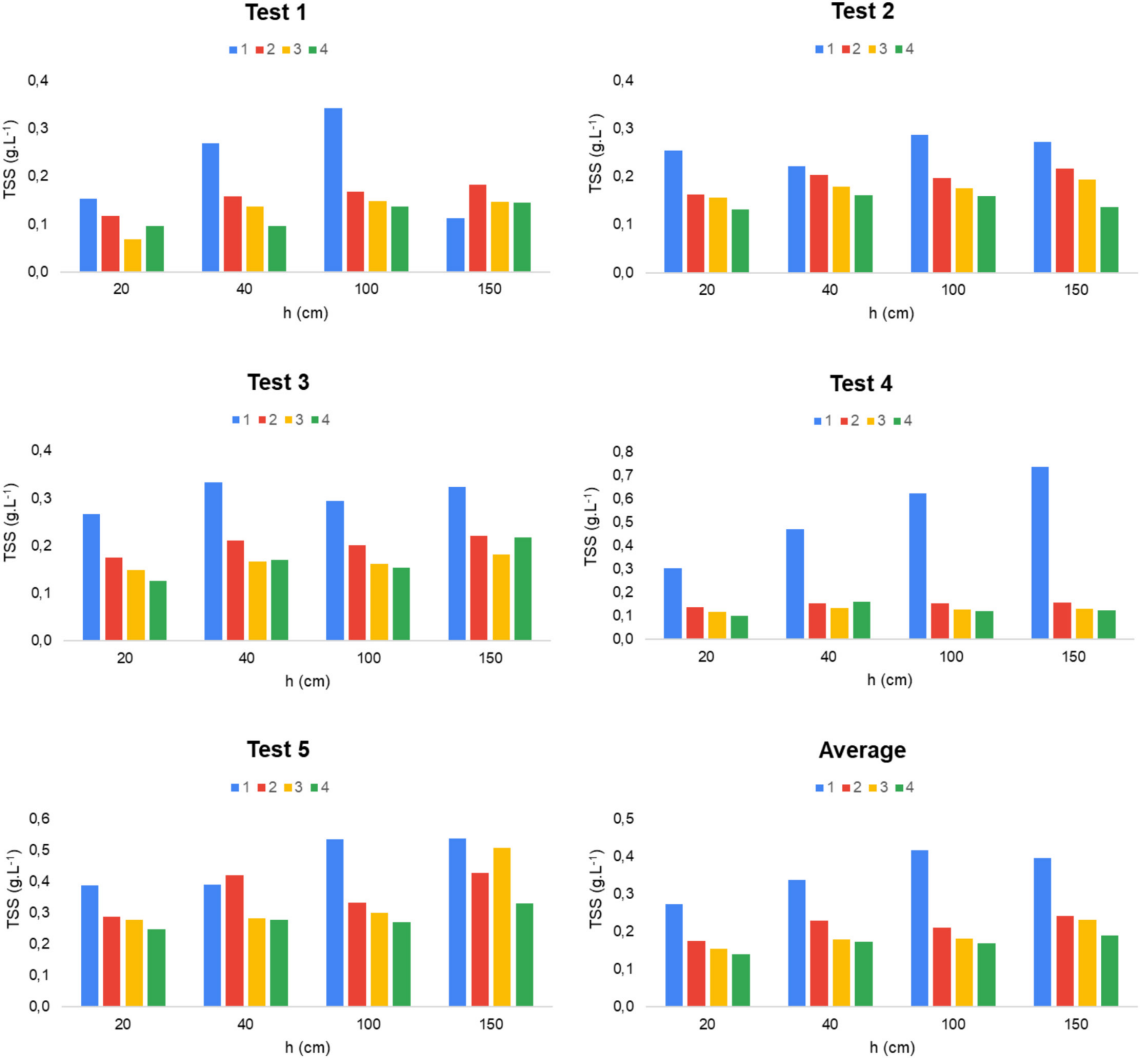
Results of the CWW settling tests, correlating TSS concentration, column depth (20, 40, 100, and 150 cm) and settling time (1, 2, 3, and 4 h).

The solids settling process for the evaluated wastewater can be observed in Figure 4, which correlates TSS concentration, settling column depth (*h*), and test duration. It should be noted that the results obtained represent the particles in their natural state, without pretreatments steps.

The results indicate significant variations in the TSS settling patterns among the performed tests, showing that the studied CWW cannot be considered homogeneous across the five tests conducted. Test 1, for example, showed the highest solids concentrations at hour 1 and at depths of 40 and 100 cm, whereas at other times and depths in this test, the TSS concentration showed little fluctuation. In Test 4, the highest solids concentration also occurred at hour 1; however, an increasing trend with depth was observed, indicating a high settling velocity. Tests 2 and 5, on the other hand, presented lower TSS settling rates as a function of time and column depth.

Considering the average result of the tests, an initial phase of extremely rapid TSS removal is observed between the first and the second hours, followed by a sharp deceleration in the settling process, at which point additional removal becomes marginal. This transition characterizes the typical kinetic signature of discrete particles settling under quiescent conditions, where solid–liquid separation occurs predominantly by the action of gravity (Mann et al. 2015; Kundu et al. 2025).

In the study conducted by Uçar (2018), a rapid process of solids sedimentation was also observed. During the settling phase, the total solids concentration decreased sharply within the first 2 h, after which it remained stable. Specifically, the concentration declined from 1054 ± 21 to 609 ± 14 mg L^−1^ after 1 h of sedimentation, corresponding to 42% solid removal.

Experimental tests in primary sedimentation tanks of industrial wastewater treatment plants have proven that the optimum retention time is equal to 2 h based on the biological oxygen demand and the TSS eliminations (Zamanikherad et al. 2023).

In the initial stage, particles with larger diameters and higher densities (which have higher settling velocities) are quickly removed, resulting in an abrupt drop in the concentration of suspended solids. As time progresses, only smaller and less dense particles remain in suspension, whose settling is slower due to the lower relative gravitational force and the greater influence of resistive forces, such as fluid viscosity (McGauhey 1956; Shajahan and Breugem 2020). This phenomenon explains the progressive reduction in the observed mass removal rate, which converges toward a stabilization plateau.

From an operational standpoint, this behavior indicates that the system's highest efficiency occurs within the first few hours, and prolonging the settling time is largely ineffective for additional solids removal. This can have direct implications for the design and optimization of treatment units. Sedimentation times of a few minutes are considered technically adequate, as they favor the reduction of the required dimensions of the grit chamber, optimizing the hydraulic design and the efficiency of the particle separation process (Vahidifar et al. 2019).

Increasing particle diameter enhances the performance of sedimentation tanks. On the other hand, the low efficiency of sedimentation tanks for fine particles can be due to the presence of a recirculation zone. This zone acts as a large eddy, allowing particles to bypass settling and reach the end of the tank. In this case, velocity variations across different sections of the tank showed that the wastewater flow connects directly from the inlet to the outlet. This phenomenon, known as short‐circuiting, can significantly reduce settling efficiency. Simulations of wastewater at different temperatures demonstrated that higher water temperatures lead to increased settling efficiency (Vahidifar et al. 2019).

The total removal efficiency (*Rt*) varied drastically between the tests, with final values ranging from 91.65% (test 1) to only 65.73% (test 2). To investigate the causes of this fluctuation, the performance of each test was correlated with antecedent rainfall conditions and initial TSS concentrations, as shown in Table 2.

**TABLE 2:** Final TSS removal as a function of initial TSS and 7‐day cumulative precipitation.

Test	7‐day cumulative rainfall (mm)	Final *Rt* (%)	TSS initial (g L^−1^)
1	16.2	91.6	1.42
2	17.0	65.7	0.41
3	24.6	73.2	0.62
4	116.1	75.3	1.91
5	11.9	66.9	1.81

The initial TSS exhibited an average of 1.23 g·L^−1^, considering all tests, reflecting the high concentration of larger‐sized solids present in the evaluated wastewater. These values fall within the range reported in the scientific literature, which varies between 0.03 and 5.8 g·L^−1^ depending on local conditions (soil, climate, urban infrastructure, etc.) (Talebzadeh et al. 2021; Kuan et al. 2022; Gharaghani et al. 2025).

The results indicate that the TSS removal efficiency by sedimentation does not depend solely on the region's rainfall levels and/or the TSS concentration of the raw effluent. Many factors clearly affect the capacity and performance of a grit chamber: surface and solids loading rates, tank type, solids removal mechanism, inlet design and location, weir placement, and loading rate (Esfahani et al. 2018).

Other factors are likely to be played in the process. The use of detergents, car shampoos, and degreasers intensifies particle dispersion and emulsifies oils, whereas the adsorption of oils and greases reduces the apparent density of the particles. Surfactants and organic matter increase the water's viscosity, and variations in pH and conductivity alter the surface charge, which can either favor or inhibit natural sedimentation (Higgins and Luthy 2006; Zhang et al. 2018; Huang et al. 2023).

The quality of the wastewater from the studied vehicle‐washing facility was evaluated in another study (Silva Júnior et al. 2025), indicating an average surfactant concentration of 69.64 (±19.04) mg L^−1^. High concentrations of surfactants, which are dispersing agents that adsorb strongly to the surface of particles, imparting a high negative surface charge and increasing the zeta potential (Sjöberg et al. 1999; Kosmulski and Mączka 2022; Jena et al. 2023). This modification promotes an electrostatic repulsion barrier that inhibits flocculation, resulting in highly stable colloidal suspensions resistant to gravitational settling. These effects can be explained by Stokes' Law, according to which the terminal settling velocity is directly proportional to the square of the particle's radius and the density difference between the particle and the fluid, and inversely proportional to the medium's viscosity (Chakraborti and Kaur 2013).

One of the limitations of the present study concerns the modeling of the sedimentation process, which is constrained by the heterogeneity of CWW. This heterogeneity requires the adoption of hybrid sedimentation models that integrate discrete and flocculent mechanisms. Such an approach is necessary because denser mineral particles (such as sand and gravel) tend to follow the behavior typical of discrete sedimentation, whereas fine and colloidal particles (clay, organic matter, and detergent residues) exhibit a greater propensity for aggregation and floc formation, characterizing flocculent sedimentation. The integration of these models enables a more accurate representation of the dynamics of suspended solids in vehicle‐washing wastewater, contributing to the proper design of treatment units and to the prediction of pollutant removal efficiency.

The results presented thus far indicate the predominance of discrete settling (type 1) over flocculant settling (type 2), which is found to be actively inhibited. This conclusion is supported by three complementary lines of argument.

As presented in Table 1, the matrix of settleable solids retained in the settling unit of the studied vehicle wash facility is composed of approximately 87% sand. Sand is a granular, inert, and noncohesive material. Its particles do not tend to aggregate in aqueous suspension, and their settling behavior is individual, determined by their intrinsic properties (Tchobanoglous et al. 2014; Von Sperling 2014).

**FIGURE 5:**
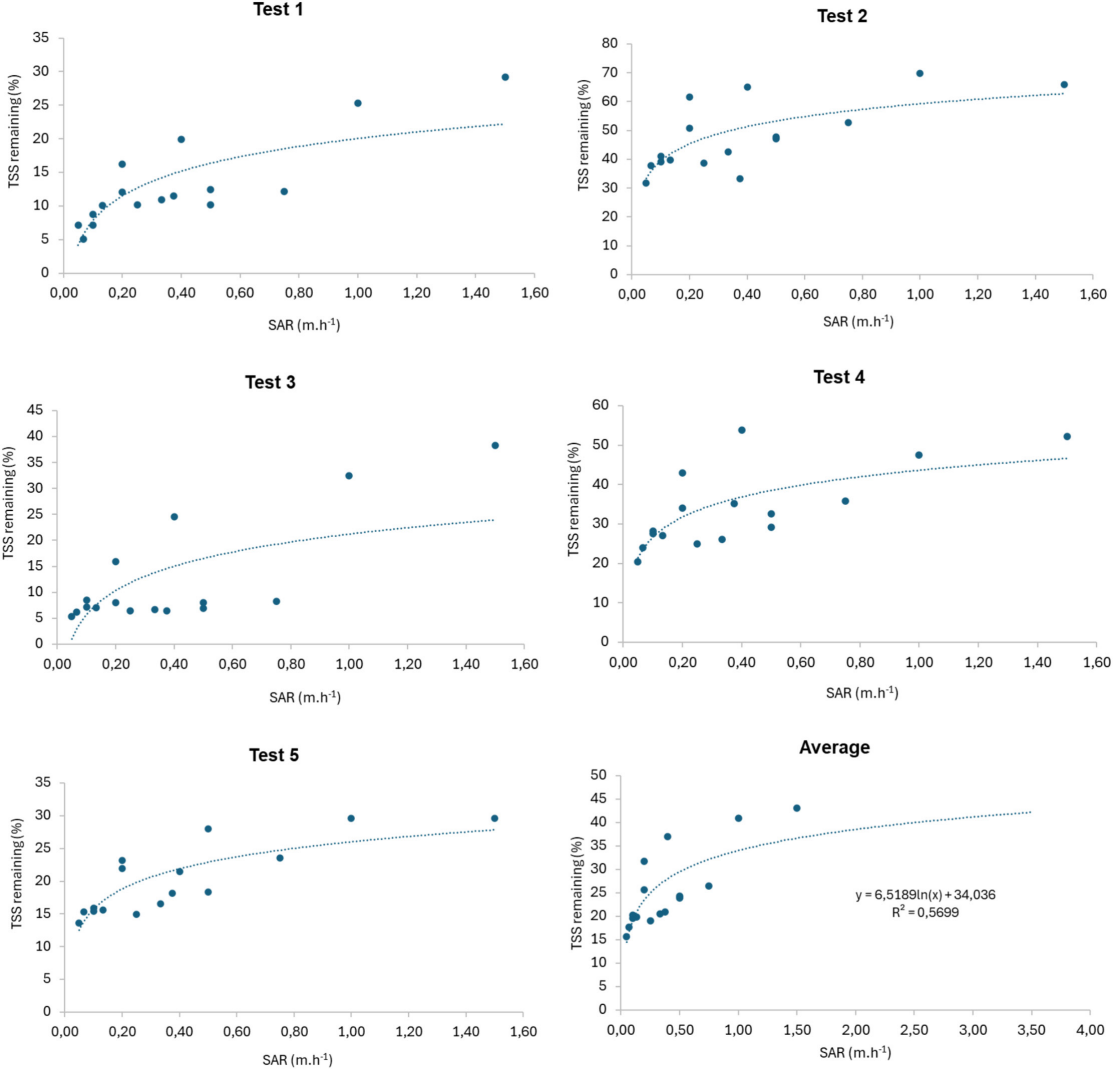
Results of the settling tests, correlating the TSS removal trend and the particle settling velocity.

Second, the kinetic evidence observed in the removal profiles of Figure 5 corroborates the discrete nature of the process. The analysis of these profiles reveals TSS removal curves with a decreasing rate over time (a typical kinetic signature of discrete settling), in which particles with the highest settling velocity are removed first, resulting in a progressive decrease in the mass removal rate (Abood et al. 2022). In none of the five tests was an induction period or an acceleration phase in removal identified, which are essential characteristics for the occurrence of flocculant settling.

Finally, the chemical evidence, which elucidates the molecular context, provides the mechanistic justification for the absence of flocculation. CWW is characterized by the presence of high concentrations of surfactants. These chemicals are potent dispersing agents, chemically designed to break up agglomerates and keep particles in suspension. Their action consists of creating electrostatic repulsion barriers around fine particles, actively inhibiting aggregation. The adsorption of these molecules onto the particle surface changes the zeta potential to highly negative values, promoting mutual repulsion that prevents the formation of flocs (Tamiazzo et al. 2015; Jena et al. 2023; Liao et al. 2024).

The experimental kinetic data, which shows nonaggregating behavior of the particles, constitutes a direct empirical validation of this chemical principle. Thus, the physical behavior observed in the settling column represents the macroscopic manifestation of interactions at the molecular level, demonstrating that the particles behave as if they were chemically inhibited from aggregating.

Several representative models of primary particle sedimentation are described in the literature. Polorigni et al. (2021), for example, evaluated primary sedimentation as a function of the composition of TSS in sewage, distinguishing between nonbiodegradable particulate organic matter, biodegradable particulate organic matter, and inorganic settleable solids. Complementarily, Vahidifar et al. (2019) investigated, through numerical simulation, the turbulent flow behavior of particles in sedimentation tanks applied to the treatment of industrial wastewater.

No specific models of solid particle sedimentation in CWW that consider its physicochemical characteristics have been identified in the scientific literature. In this context, future studies should focus on such investigations to deepen the understanding of the processes involved and to support the development of more representative models for the design and optimization of treatment units.

The design of settling units must be carried out considering the most critical conditions (typical of the rainy season, with a high TSS load and the presence of oils, greases, and surfactants) to ensure consistent performance and protect subsequent treatment stages, even when faced with significant variations in CWW quality. The unit must be designed to remove particles with a specific cut‐off diameter. A commonly adopted value for grit chambers is the efficient removal of particles with a diameter equal to or greater than 0.2 mm (Tchobanoglous et al. 2014; Von Sperling 2014).

**FIGURE 6:**
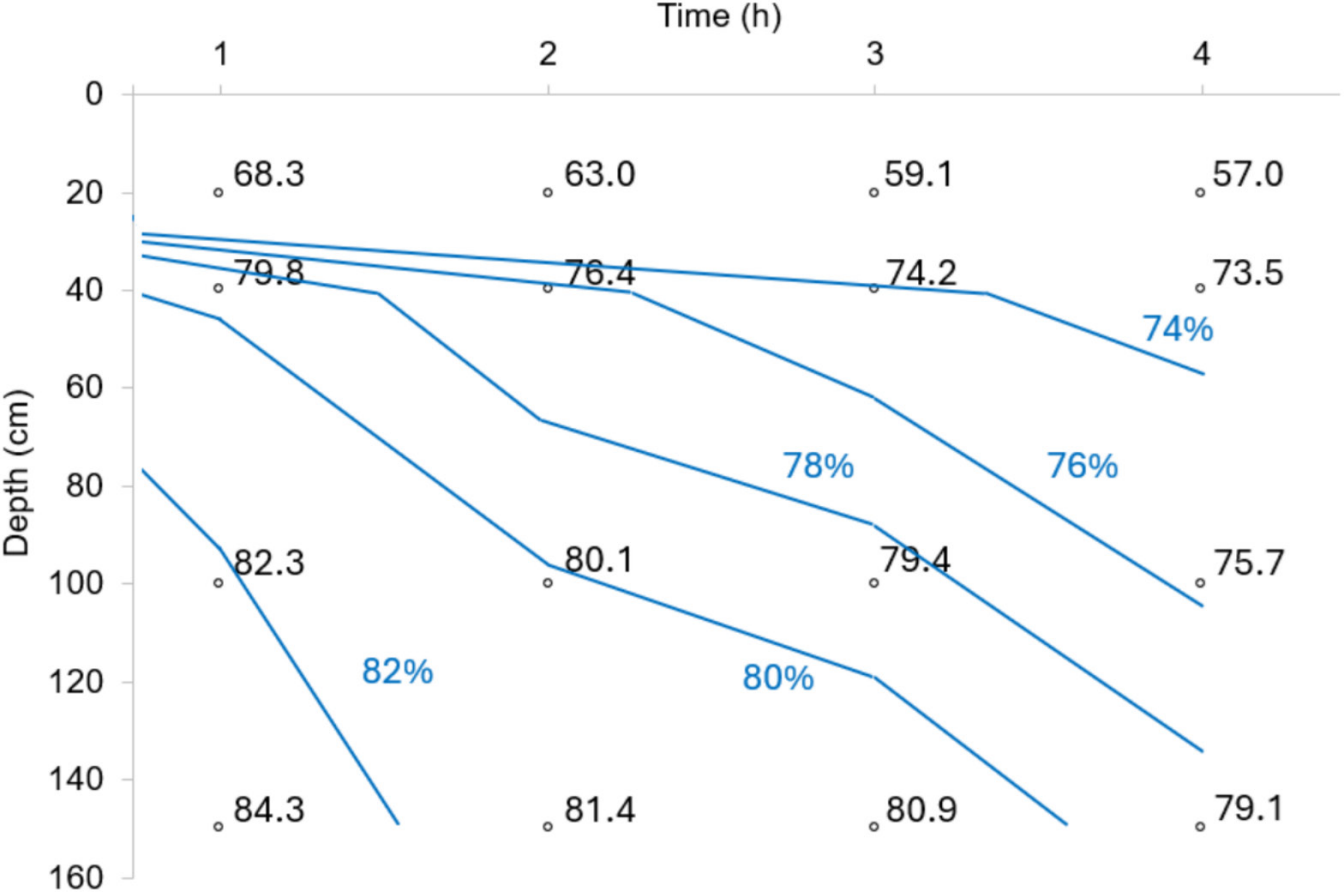
Iso‐removal curves of TSS (%) considering the average values of the tests performed.

Evaluating the average behavior of the tests performed (Figure 5), and iso‐removal curves (Figure 6), it is observed that the SAR shows little variation in TSS removal efficiency in the evaluated range of 0.5 to 1.5 m·h^−1^. It is also observed that HRTs shorter than 1 h prove to be suitable for the sedimentation process in CWW treatment.

Thus, aiming to reduce the size of the settling units, the use of a SAR of 1.5 m·h^−1^ is suggested, which corresponds to an average TSS removal efficiency of approximately 80%. It is recommended that values close to this be used as a reference for the design of CWW grit chamber.

For example, considering the evaluated car wash business, with a daily flow of 30.6 m^3^ (operating for 10 h), a 2.04‐m^2^ grit chamber will be necessary to remove 80% of TSS with a SAR of 1.5 m·h^−1^. Although higher efficiencies can be obtained by reducing the SAR, such a measure implies a proportional increase in the unit's surface area.

Aiming to reduce the size of the sand removal unit, the logarithmic projection (*R* ^2^ = 0.57) of remaining TSS = 6.5 × *ln* (SAR) + 34 can be used, implying that increasing the SAR (and consequently reducing the surface dimensions of the grit chamber) will also decrease the TSS removal from the CWW. For solid particles present in domestic sewage, generally with an average size equal to or greater than 0.21 mm, a SAR between 60 and 78 m·h^−1^ is suggested for the design of grit chamber (Jordão and Pessoa 2011; Tchobanoglous et al. 2014). Applying SAR = 69 m·h^−1^ in the presented projection, a TSS removal efficiency of only 61.5% is obtained, despite the reduced dimensions of grit chambers.

The results obtained from the CWW sedimentation tests should be regarded as independent reference values, and the extrapolation of SAR parameters used for domestic sewage is not recommended. The adoption of specific design parameters for CWW provides greater reliability in the sizing of treatment units, supporting both operational efficiency and the economic viability of the system. Moreover, removal efficiencies of TSS exceeding 80% facilitate the operation and maintenance of subsequent treatment units.

Although domestic sewage has relatively stable and predictable characteristics (already widely addressed in scientific literature) (Jordão and Pessoa 2011; Tchobanoglous et al. 2014; Von Sperling 2014), CWW has great variability depending on the type of soil adhering to the vehicles, the frequency of washing, the chemical products used, and even the local climatic conditions (Monney et al. 2020; Espinoza‐Montero et al. 2023; Talebzadeh et al. 2021; Cuput et al. 2024). This variability reinforces the need for specific tests because the adoption of domestic sewage parameters could lead to design errors, resulting in undersized units with low solids removal efficiency.

Although the wastewater sampling procedure was conducted using a composite method, with a collection period of 35 min, it is likely that the daily concentrations of TSS were not fully representative. This limitation arises because vehicles originating from different urban sectors, with varying degrees of particulate accumulation, arrive at washing facilities and generate effluents characterized by spatial and temporal heterogeneity in TSS loads. Consequently, further investigations are required to assess pollutant variability in vehicle‐washing wastewater, considering hydraulic detention time, climatic conditions, soil typology, urban infrastructure (e.g., pavement type), and population behavioral patterns.

The presence of a significant and variable nonsettleable fraction, which can reach about 20% of the total solids load (for SAR of 1.5 m·h^−1^), demonstrates that simple sedimentation is insufficient for the CWW to meet environmental quality standards. The residual turbidity, composed of chemically stabilized colloidal particles, cannot be removed through operational adjustments in the settling unit, such as increasing the detention time or the tank volume.

Therefore, the implementation of a complementary treatment stage is necessary. Processes such as filtration, coagulation‐flocculation, electrocoagulation, flotation, membrane separation, and advanced oxidation are widely discussed in scientific literature as viable alternatives for the removal of this residual fraction (Kuan et al. 2022; Espinoza‐Montero et al. 2023; Obura et al. 2023; Silva Júnior et al. 2025).

## 4 Conclusion

The analysis of settleable solids from the carwash wastewater treatment system revealed a predominance of the sand fraction (D_90%_ = 1.1 mm), with an average of 87.44%. This characteristic confirms the coarse texture of the retained material and its high sedimentation velocity during the first hour. However, the settling tests for TSS demonstrated a highly variable efficiency, which did not directly correlate with the initial solid concentration or with rainfall conditions. Results indicated the need for hybrid sedimentation models to adequately represent TSS sedimentation for CWW.

The adoption of a SAR of 1.5 m·h^−1^ is suggested, which corresponds to an average TSS removal efficiency of approximately 80%. Consequently, simple sedimentation, although fundamental as a pretreatment step for the removal of coarse particles, proves to be insufficient to ensure that the final effluent meets environmental quality standards. The existence of a significant nonsettleable fraction, which can reach up to 20% of the TSS load, highlights the need to implement complementary treatment stages.

## Author Contributions

**João Paulo Cruvinel Miranda:** investigation, writing – original draft, methodology. **Antônio Alves Martins:** investigation, validation. **Andriane de Melo Rodrigues:** writing – review and editing, investigation, data curation. **Celsio Assane:** investigation, methodology, writing – original draft. **Édio Damásio da Silva Júnior:** conceptualization, writing – review and editing, project administration, supervision.

## Funding

This work was supported by Fundação de Amparo à Pesquisa do Estado de Goiás (202410267000957).

## Conflicts of Interest

The authors declare no conflicts of interest.

## Data Availability

The data that support the findings of this study are available from the corresponding author upon reasonable request.
